# Salzburg Database of Polygonal Data: Polygons and Their Generators

**DOI:** 10.1016/j.dib.2020.105984

**Published:** 2020-07-08

**Authors:** Günther Eder, Martin Held, Steinþór Jasonarson, Philipp Mayer, Peter Palfrader

**Affiliations:** Universität Salzburg, FB Computerwissenschaften, Salzburg, Austria

**Keywords:** Polygons, Generators, Database, Pseudo-random, Monotone, Star-shaped

## Abstract

The Salzburg Database is a repository of polygonal areas of various classes and sizes, with and without holes. Positive weights are assigned to all edges of all polygons. We introduce this collection and describe the generators that produced its polygons. The source codes for all generators as well as the polygons generated are publicly available.

##    Specifications table

**Table:** 

Subject	Computer Graphics and Computer-Aided Design

Specific subject area	The Salzburg Database is a repository of polygonal areas of various classes and sizes, with and without holes. Positive weights are assigned to all edges of all polygons.
Type of data	Text files and C/C++ codes used to generate the data.
How data were acquired	All polygonal data was generated by our codes at the University of Salzburg, Salzburg, Austria.
Data format	Raw data in GraphML [1] format (for the polygons) and C/C++ codes (for the generators).
Parameters for data collection	Sample data is described; the full set of thousands of polygons is available in the repository.
Description of data collection	Our C/C++ codes were used to generate the data; all codes are available in the repository.
Data source location	All data was generated at the University of Salzburg, Salzburg, Austria.
Data accessibility	Data is stored in two repositories. Repository name for polygonal data: Salzburg Database of Geometric Inputs. Direct URL to data:. See also https://sbgdb.cs.sbg.ac.at. Repository name for the codes: Computational Geometry and Applications Laboratory. Direct URL to the codes: https://github.com/cgalab.

## Value of the data

• An important part of software development is testing the correctness and evaluating the performance of an algorithm’s implementation. Ideally, one would run one’s code on data of practical relevance. However, when working on implementations of geometric algorithms it often is next to impossible to obtain enough practically relevant inputs. Then the second-best choice is to run an algorithm for a reasonably large number of “random” inputs. Subjecting the code to inputs of different characteristics is important since this may help to trigger different execution paths. Similarly, a large range of input sizes is needed to obtain insights in the actual runtime and memory consumption.
• Researchers and developers working on implementations of geometric algorithms in both academia and industry will benefit from this data if they need polygons to test their codes.
• The availability of this data permits future experimental studies (such as performance evaluations) that require a large number of polygonal datasets.
• Random polygons are used in various other fields outside of computer science.
• Users of our polygonal data can easily generate additional data of their own because we provide the source codes of all our generators.

## 1 Data Description

The Salzburg Database provides a repository of polygonal data. It contains simply-connected and multiply-connected polygonal areas in two dimensions. Every polygon has positive weights assigned to its edges. These weights can be used to test codes that operate on weighted polygonal input, such as for computing weighted straight skeletons. Of course, these weights can also be regarded as weights assigned to the vertices of the polygon, by, e.g., taking the weight of an edge as the weight of its start vertex.

We use GraphML [1] as file format for our polygonal data. This file format is extensible. Hence, we could also add explicit vertex-weights and other information such as edge or vertex colorings in the future.

Our database can be used freely and is provided via direct download from https://sbgdb.cs.sbg.ac.at or git in combination with git-annex. (See https://git-annex.branchable.com/.) It is also hosted on Zenodo, https://zenodo.org/. See doi:10.5281/zenodo.3784788 for a persistent link. Perhaps even more important is the fact that the source codes for all generators used to generate our polygonal data are available on GitHub and can be used freely under the GPL(v3) license: See https://github.com/cgalab.

## 2 Experimental Design, Materials and Methods

### 2.1 Triangulation Perturbation

Our implementation Fpg is motivated by an approach originally proposed by O’Rourke and Virmani [2]: They start with a regular polygon P and then translate its vertices while maintaining the polygon’s simplicity. A direction and speed are chosen at random and assigned to each vertex of P. Then the vertices of P are processed consecutively. A single vertex is moved one “time unit” as long as P remains simple, otherwise that move is omitted and a new random velocity is chosen for the next round. O’Rourke and Virmani [2] suggest to use several hundred translations per vertex.

As vertices can also move in an outward direction, a domain is defined which has to contain P. We use a large rectangle to limit the outward movement of the vertices.

**Fig. 1:**
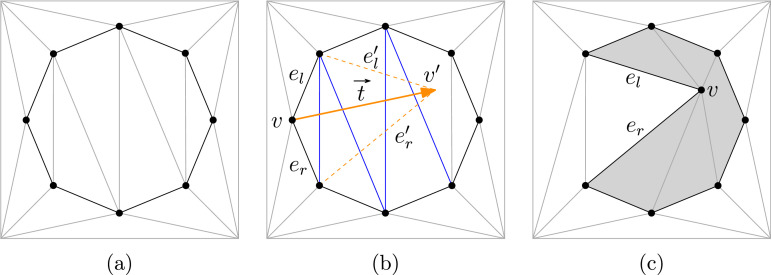
(a) Triangulation of the start polygon and its domain; (b) Translation of vertex *v*; (c) The polygon after the translation of *v*.

Maintaining the simplicity of P during the vertex translations can be an expensive task if carried out naïvely. We utilize a triangulation of the interior and the exterior of P to simplify intersection tests while moving a polygon vertex; cf. Fig. 1a. Let *v* denote a boundary vertex of P that we want to translate and let *e_l_* and *e_r_* denote its two incident edges. In practice, a randomly chosen translation vector $\vec{t}$ tends to violate the simplicity of P, with high probability, which leads to a bad performance. Therefore, we choose a random direction for $\vec{t}$ first. Then the length of $\vec{t}$ is generated from a normal distribution using parameters suitable to the local environment around *v*, in the chosen direction. Experiments show that such an approach for choosing translation vectors produces only few invalid translations.

After translating *v* by $\vec{t},$ we obtain *v*′ and the edges $e_{l}^{\prime }$ and $e_{r}^{\prime },$ respectively. Our intersection test involves checking all triangles pierced by $e_{l}^{\prime }$ or $e_{r}^{\prime }$. If all triangle edges intersected by $e_{l}^{\prime }$ and $e_{r}^{\prime }$ are interior or exterior diagonals then we change *v* into *v*′ in P. Additionally, we may have to modify the triangulation by checking the triangles intersected by the modified edges as well as the triangles incident at *v*. If we cross a polygon edge then we reject $\vec{t}$ as translation vector and restart the process. See Figure for an illustration of this process.

Fpg starts from a regular polygon where a triangulation, in- and outside, is trivially obtained. To speed up the generation of large polygons, instead of starting with a large regular polygon, Fpg can start with a smaller one, and then “grow” this polygon by repeatedly splitting random edges. The additional vertex introduced by the split is then translated to avoid collinearities.

**Fig. 2:**
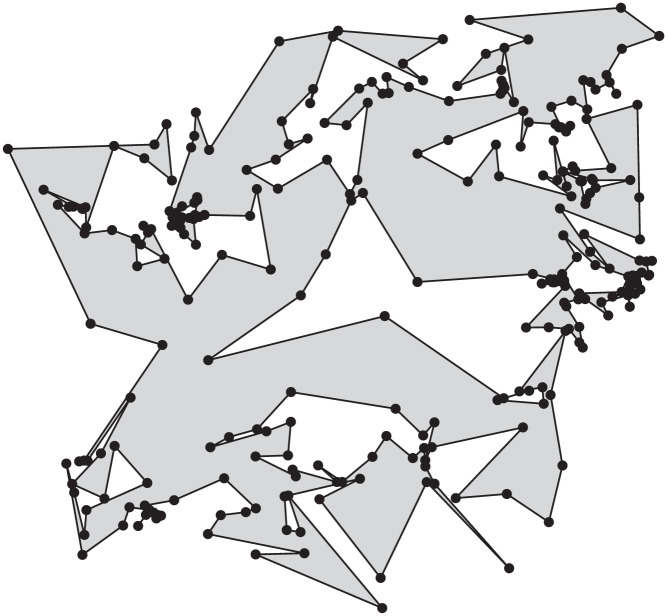
Polygon exhibiting clustering due to the selection of edges uniformly at random in the subdivision step.

If we pick edges uniformly at random then we see clusters of many short edges and a few very long edges. Presumably this is due to the fact that areas with short edges are more likely to get extra vertices than areas of the same size which contain (fewer) long edges; cf. Fig. 2. To avoid this clustering, we pick edges randomly weighted by their length.

**Fig. 3:**
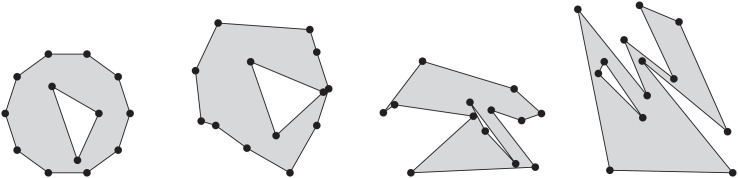
Polygons generated by Fpg after 1, 8, 50, and 500 iterations without edge-subdivision.

**Fig. 4:**
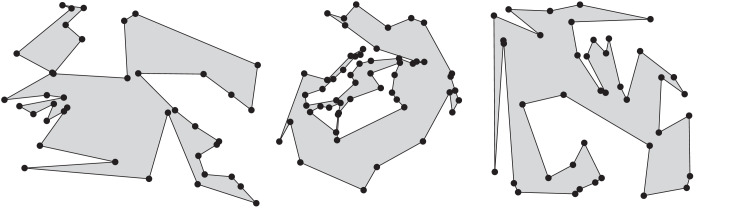
Left-to-right: A polygon and a polygon with holes computed by Fpg, and a polygon generated by Spg. All polygons have of 40 vertices.

Furthermore, Fpg is capable of generating polygons with holes. Since P is regular at the beginning, we can trivially place regular holes inside P as well. The process described above works also for this setting, as the intersection tests hinge on the triangulation. In Fig. 3 we illustrate the evolution of a polygon computed by Fpg. The polygon has 10 vertices, with a triangular hole formed by three additional vertices. The first two images in Fig. 4 are the result of Fpg using edge-subdivision; the second image depicts a polygon with holes.

### 2.2 Combining Line Sweep and 2-Opt Moves

Our generator Spg constructs a simple polygon P on a given point set *S* in the plane. (Such a point set can be generated randomly or specified by a user.) Initially, Spg creates a polygon by choosing a random permutation of the input vertices. This initial polygon contains, with high probability, self-intersections. Therefore, a line sweep is applied to identify intersecting pairs of edges, followed by local modifications which remove these intersections.

To identify pairs of edges that intersect we use the classic Bentley-Ottmann algorithm [3]. We sweep from left to right, thereby maintaining a sorted set of edges that intersect the sweep-line. The input vertices comprise the event points of the line sweep. During the sweep, at vertex *v_i_*, we have to modify the sweep-line status by removing and/or adding the edges incident at *v_i_*. Additionally, at every event point, we have to verify that any newly added edge is not intersecting its neighbors in the status. In case a pair of edges does intersect, we have to resolve that intersection before we carry on with the sweep.

We resolve intersections by applying so-called *2-opt* moves. A 2-opt move replaces the edges $e_{1}=\bar{v_{1}v_{2}}$ and $e_{2}=\bar{v_{3}v_{4}}$ by the edges $e_{1}^{\prime }=\bar{v_{1}v_{3}},e_{2}^{\prime }=\bar{v_{2}v_{4}}$. (Note that the polygon boundary becomes disconnected if the 2-opt move connects the wrong vertex pairs.) As we apply 2-opt moves during the line sweep to resolve intersections, we may introduce new intersections. However, a key property of the 2-opt move is that it decreases the length of the polygon (if not all points are collinear). This guarantees that we will eventually arrive at a polygon that is simple if we apply 2-opt moves repeatedly to resolve intersections. A result by van Leeuwen and Schoone [4] tells us that we need at most $O(n^{3})$ 2-opt moves.

We implemented and tested three variants of the line sweep. They differ mainly in how they proceed after finding and resolving an intersection:

(a) After a 2-opt move is carried out, we simply continue with the line sweep. After arriving at the right-most vertex we restart the line sweep at the left-most vertex. The sweep is repeated until all intersections are resolved.
(b) After a 2-opt move, we test and resolve all intersections at the current sweep-line position, before carrying on. Again, at the right-most vertex we restart until all intersections are resolved.
(c) After a 2-opt move, we reverse the sweep direction to deal with possibly new edge intersections. We resume our rightwards sweep at the left-most vertex affected by the 2-opt move.

The last image in Fig. 4 was generated by Spg on a point set of 40 vertices using variant (a).

Note that collinear edges need special care because a 2-opt move will not always result in a shortening of the perimeter of the polygon. If intersecting collinear edges are detected then we remove these edges and sort the respective collinear vertices. Then we connect the vertices by edges in consecutive order, i.e., form a chain of non-overlapping collinear edges. This guarantees that the perimeter of the polygon decreases also in the case of collinear vertices.

### 2.3 SRPG

**Fig. 5:**
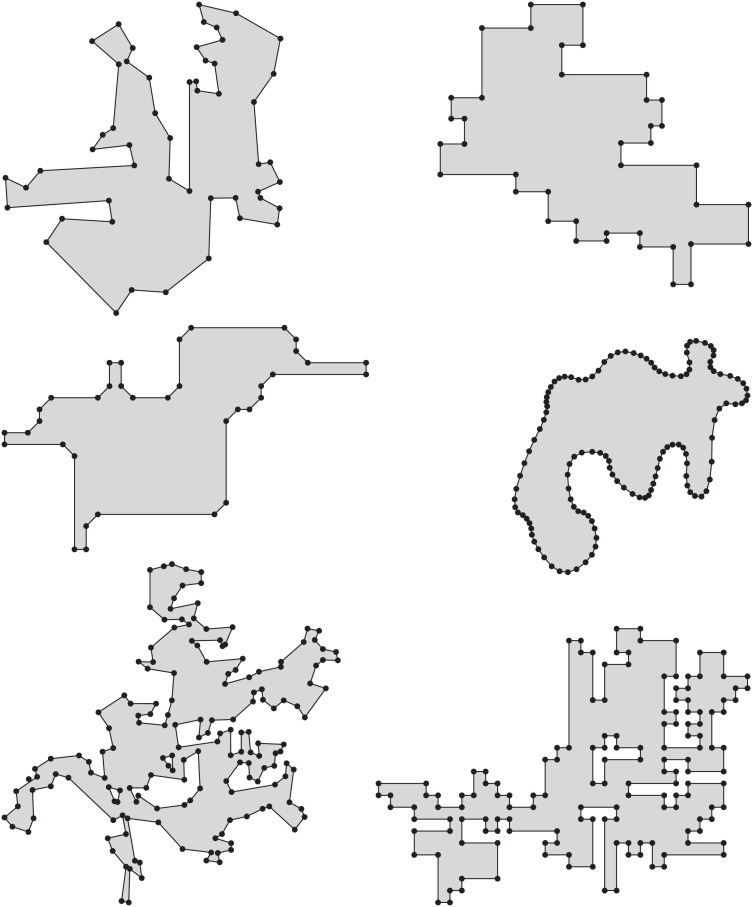
Samples of a random, an orthogonal, an octagonal, and a smoothed polygon generated by Srpg, as well as a random and a grid-aligned orthogonal polygon with holes.

Srpg generates simply-connected and multiply-connected polygonal areas by means of a regular grid that consists of square cells. Given two integer values, *a* and *b*, Srpg generates a grid of size *a* times *b*. By default Srpg then generates orthogonal polygons on this grid. An additional parameter *p*, between zero and one, leads to a smaller or larger number of vertices in the produced polygon. Srpg is able to produce octagonal polygons by cutting off corners with ±45^∘^ diagonals during the construction. Cutting corners repeatedly, without the diagonal restriction, yields an approximation of a smooth free-form curve. Additionally, Srpg can apply perturbations in order to generate polygons with axes-parallel edges whose vertices do not lie on a grid, or to generate polygons whose edges (in general) are not parallel to the coordinate axes. See Fig. 5 for some sample polygons.

### 2.4 RPG

**Fig. 6:**
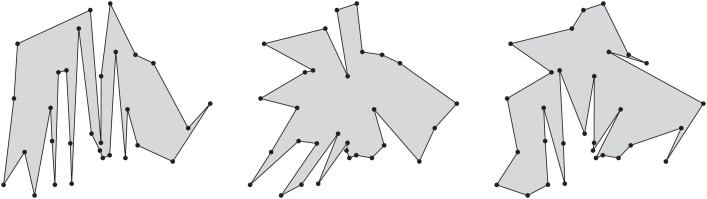
In left-to-right order, an *x*-monotone, a star-shaped, and a simple polygon computed by Rpg on the same set of 30 vertices.

Auer and Held [5] first described Rpg more than twenty years ago. Rpg supports various heuristics to generate “random” polygons for a given set of vertices. In particular, it is able to produce star-shaped polygons uniformly at random. Furthermore, it generates *x*-monotone polygons uniformly at random, based on the algorithm by Zhu et al. [6]. We have resurrected this code and updated it to compile on modern platforms, thus meeting requests voiced by several colleagues. A recent extension of Rpg also supports the generation of polygons with holes. See Fig. 6 for examples of some polygons generated by Rpg.

### 2.5 Additional Generators

**Fig. 7:**
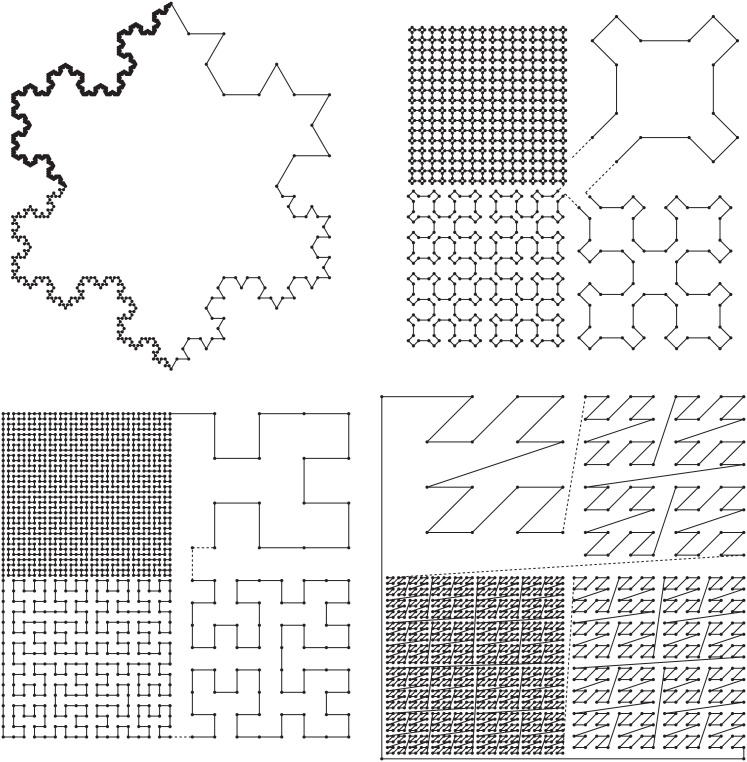
The curves of Koch, Sierpinski, Hilbert, and Lebesgue, in reading order. Each figure is partitioned into four quadrants with portions of the curve shown at different orders.

Our repository also contains codes to produce well-known polygons such as the Koch snowflake (also in a nested variant), the Sierpinski curve, and closed variants of the Hilbert and Lebesgue curves; see Fig. 7.

## Declaration of Competing Interest

The authors declare that they have no known competing financial interests or personal relationships which have, or could be perceived to have, influenced the work reported in this article.

## Appendix A Supplementary materials

Supplementary Data S1 Supplementary Raw Research Data. This is open data under the CC BY license http://creativecommons.org/licenses/by/4.0/ Supplementary Data S1

## References

[1] Brandes U., Eiglsperger M., Herman I., Himsolt M., Marshall M.S. (2001). GraphML Progress Report Structural Layer Proposal. Proceedings of the 9th International Symposium on Graph Drawing.

[2] O’Rourke J., Virmani M. (1991). Generating Random Polygons. Technical Report.

[3] Bentley J.L., Ottmann T.A. (1979). Algorithms for Reporting and Counting Geometric Intersections. IEEE Transactions on Computers.

[4] van Leeuwen J., Schoone A.A., Mühlbacher J. (1982). Untangling a Travelling Salesman Tour in the Plane. Proc. 7th Conference Graph-theoretic Concepts in Computer Science (WG’81).

[5] Auer T., Held M. (1996). Heuristics for the Generation of Random Polygons. Proceedings of the 8th Canadian Conference on Computational Geometry (CCCG).

[6] Zhu C., Sundaram G., Snoeyink J., Mitchell J. (1996). Generating Random Polygons with Given Vertices. Computational Geometry: Theory and Applications.

